# Magnetic Field Enhanced Superconductivity in Epitaxial Thin Film WTe_2_

**DOI:** 10.1038/s41598-018-24736-x

**Published:** 2018-04-25

**Authors:** Tomoya Asaba, Yongjie Wang, Gang Li, Ziji Xiang, Colin Tinsman, Lu Chen, Shangnan Zhou, Songrui Zhao, David Laleyan, Yi Li, Zetian Mi, Lu Li

**Affiliations:** 1 Department of Physics, University of Michigan, Ann Arbor, MI 48109 USA; 2 Department of Electrical and Computer Engineering, McGill University, Montreal, Quebec, H3A 0E9 Canada; 3 Department of Physics and Astronomy, Johns Hopkins University, Baltimore, MA USA

## Abstract

In conventional superconductors an external magnetic field generally suppresses superconductivity. This results from a simple thermodynamic competition of the superconducting and magnetic free energies. In this study, we report the unconventional features in the superconducting epitaxial thin film tungsten telluride (WTe_2_). Measuring the electrical transport properties of Molecular Beam Epitaxy (MBE) grown WTe_2_ thin films with a high precision rotation stage, we map the upper critical field *H*_*c*2_ at different temperatures *T*. We observe the superconducting transition temperature *T*_*c*_ is enhanced by in-plane magnetic fields. The upper critical field *H*_*c*2_ is observed to establish an unconventional non-monotonic dependence on temperature. We suggest that this unconventional feature is due to the lifting of inversion symmetry, which leads to the enhancement of *H*_*c*2_ in Ising superconductors.

## Introduction

Superconductivity generally competes with magnetic fields. Based on thermodynamics, an applied magnetic field usually suppresses superconductivity by destroying the underlying electron pairing in the superconducting state^1^. This pair breaking principle has been confirmed in thousands of superconductors. However, this simple principle may be invalid when strong spin-orbit-coupling or symmetry protection brings novel physics to bear on the superconducting state^2^. As a result, the superconducting transition temperature (*T*_*c*_) is expected to be enhanced by magnetic fields in the finite momentum pairing system with strong Rashba-type spin orbit coupling^3^, non-centrosymmetric superconductors^4^, in topological superconductors^5^, and notably in the unconventional Ising superconductors based on atomic layered transition metal dichalcogenides (TMD)^6^. In particular monolayered TMD hosts unique valley and spin degrees of freedom, which leads to a number of novel phenomena^7–16^ due to their unique non-centrosymmetric crystal structure. The unique lifting of the inversion symmetry leads to Ising superconductivity in MoS_2_ and NbSe_2_^6,15–19^, which have an upper critical field *H*_*c*2_ as high as 5 to 10 times larger than the paramagnetic Pauli limit *H*_*p*_. The notable prediction of the theory underlying Ising superconductivity is the non-monotonic temperature (*T*) dependence of *H*_*c*2_ in the ground state^6^ due to the competition between Ising and Rashba type spin-orbit coupling. The former interaction enhances the superconductivity while the latter suppresses superconductivity. Thus, TMD materials are expected to show both non-monotonic *H*_*c*2_ predicted at low temperature and *T*_*c*_ enhancement by the magnetic field due to the non-centrosymmetric crystal structure. However, while high *H*_*c*2_/*H*_*p*_ has been observed in MoS_2_ and NbSe_2_, neither non-monotonic *H*_*c*2_ nor *T*_*c*_ enhancement by magnetic field has been observed so far. Therefore, direct observation of these features is important to understand the Ising superconductivity. Furthermore, both MoS_2_ and NbSe_2_ have the hexagonal 2 H crystal structure, and the system becomes non-centrosymmetric only when the thin film consists of odd atomic layers. It is still unknown if Ising pairing can exist in a different crystal structure, especially when the bulk crystal itself is non-centrosymmetric.

The best candidate to search for non-centrosymmetric Ising superconductivity is superconducting tungsten telluride (WTe_2_). Many unique topological phases are predicted in this family of TMDs, such as quantum spin Hall effect in the monolayered WTe_2_ film^20^, and type-II Weyl semimetal in MoTe_2_ and WTe_2_^21^. The type-II Weyl state was further confirmed by a number of photoemission studies in both Te-based TMD materials and in related materials^22–28^. These features are deeply connected to its unique T_*d*_ crystal structure, the bulk non-centrosymmetric structure. Furthermore, a giant magnetoresistance was also observed in WTe_2_^29^. A superconducting state has been observed under high pressure in WTe_2_^30,31^ and in ambient pressure in MoTe_2_^32^. The interplay between the type-II semimetal phase and superconductivity could give rise to many unconventional features. In this letter, we not only observed the highest *H*_*c*2_/*H*_*p*_ (10) in TMD materials, but also discovered two unique unconventional features in superconducting WTe_2_ thin films — the magnetic-field-enhancement of superconductivity and non-monotonic *H*_*c*2_ as a function of temperature.

To study the ground state superconducting pairing requires the growth of thin films. The film thickness provides a geometrical constraint that is smaller in our thin-film samples than the bulk coherence length. This removes the orbital effects of an in-plane magnetic field that would otherwise suppress the superconductivity in the bulk, thereby realizing the novel conditions for Ising superconductivity. This has been demonstrated by the observation of 2D Ising superconductivity in mechanically exfoliated NbSe_2_ and MoS_2_^6,15–19^, showing high *H*_*c*2_/*H*_*p*_. However, TMD monolayered devices are realized by pioneering work in thinning layered TMD materials into single atomic layers using mechanical exfoliation^7^, chemical vapor deposition^33^, and epitaxial growth^34–37^. The mechanical exfoliated WTe_2_ or MoTe_2_ devices are generally micron sized and their electronic mobility in the monolayer limit is generally too low to host an interesting ground state. In our paper, we report the first Molecular Beam Epitaxy (MBE) growth of thin WTe_2_ films.

## Results

Thin films of WTe_2_ with a thickness of 5.5, 7, 10 and 14 nm were grown on *c*-Al _2_O_3_ (0001) substrate using a Veeco Genxplor MBE growth system (see the supplement).The scanning probe microscopy (SPM) image of the WTe_2_ film exhibits smooth and continuous surface morphology. The surface roughness is estimated to be ∼0.22 nm, without the presence of any sharp edges, wrinkles, or discontinuities. The stoichiometric analysis was performed by high-resolution X-ray photoelectron spectroscopy immediately after growth. The shape and position of the core-level W-4d and Te-3d peaks are consistent with previous studies of WTe_2_ crystal structures^35,38^. The presence of W and Te oxidation peaks were not observed, confirming the high purity of epitaxial WTe_2_ thin films. The ratio of W and Te was measured to be 1:1.93, suggesting the formation of nearly stoichiometric WTe_2_. They were uniformly formed on a sapphire substrate with precise thickness control. We observed two-dimensional superconductivity in the ground state and a Berezinskii-Kosterlitz-Thouless (BKT) transition (see the supplement).

**Figure 1:**
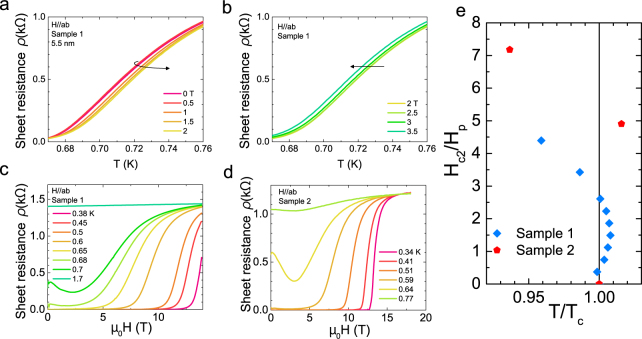
Field-induced enhancement of *T*_*c*_ at low in-plane field and high temperature. (**a**,**b**) The temperature dependence of the 5.5 nm WTe_2_ Sample 1 (*T*_*c*_ 0.71 K) film sheet resistance, *ρ*, at fixed fields. The black arrow marks the direction of the increase of in-plane magnetic fields. In Panel a, the superconducting transition shifts to higher *T* as the in-plane field increases to 2 T, whereas at higher field, the transition shifts to lower *T*. Combining panel a and b shows the transition temperature *T*_*c*_ gets enhanced at the finite in-plane magnetic field. (**c**) The magnetic field *H* dependence of 5.5 nm WTe_2_ film Sample 1. At around *T*_*c*_, this sample shows strong negative magnetoresistance. (**d**) Similar negative magnetic field *H* dependence of *R* is observed in 5.5 nm WTe_2_ film Sample 2 (*T*_*c*_ 0.64 K). (**e**) The temperature dependence of in-plane upper critical field for 5.5 nm WTe_2_ thin film around *T* = *T*_*c*_. Field-induced enhancement of *T*_*c*_ shows a maximum of 0.8% in sample 1 (*H*_*p*_ = 1.31 T) and 1.6% in sample 2 (*H*_*p*_ = 1.21 T).

The resistivity data of 5.5 nm WT _2_ films at around the critical temperature is shown in Fig. 1. Specifically, Fig. 1(a) and (b) give the resistivity data from sample 1 as a function of temperature at the fixed magnetic field around critical temperature. The *T*_*c*_ clearly increases with magnetic field up to 2 T (Fig. 1(a)), then starts to decrease with higher field (Fig. 1(b)). The *T*_*c*_ enhancement is about 10 mK at 2 T. Even larger magnetic-field-enhancement of *T*_*c*_ was observed in other samples, as shown in Fig. 1(e) for Sample 2, corresponding to 1.6% enhancement. The large negative magnetoresistance around *T*_*c*_ is connected to the observed enhancement of *T*_*c*_ as shown in Fig. 1(c) and (d). Thicker samples also show similar negative magnetoresistance at around *T*_*c*_ (see supplement). Figure 1(e) shows a phase diagram of *H*_*c*2_ versus *T*/*T*_*c*_ for two 5.5 nm WTe _2_ thin film samples around *T* = *T*_*c*_ with the magnetic field applied in-plane. For sample 1, the *H*_*c*2_/*H*_*p*_ vs *T*/*T*_*c*_ clearly shows the enhancement of *T*_*c*_ with increasing magnetic field. Sample 2 shows a larger enhancement of 1.6% at 3 T. The difference of enhancement between samples may originate from the thickness difference due to the 10% error of our thickness measurement. As shown later, *T*_*c*_ of sample 2 (0.64 K) is lower than that of sample 1 (∼0.71 K), indicating slightly thinner film thickness.

For comparison, *H*_*c*2_ is normalized by Pauli limit *H*_*p*_. In a BCS superconductor, the Pauli paramagnetic limit (*H*_*p*_) is the magnetic field value where the paramagnetic Zeeman energy is the same as the superconducting condensation energy. This limit generally is the upper bound for the in-plane *H*_*c*2_ because there are no orbital degrees of freedom in a two-dimensional system, so only the spins should determine the superconducting property^39,40^. In the weakly coupled limit, the BCS superconductor condensation energy is 3.52*k*_*B*_*T*_*c*_. Thus in units of tesla *μ*_0_*H*_*p*_ = 1.84 * *T*_*c*_ where *T*_*c*_ has units of Kelvin^1^. Moreover, in the high field normal state *R* becomes almost constant within the measurement noise limit (see also supplement). Thus, we define the upper critical field *H*_*c*2_ as 50% of the constant resistivity value at high fields).

Note that the smaller resistivity at finite fields is not due to the negative magnetoresistance from the normal state, but rather the enhancement of superconductivity. First, since the magnetic field is applied in-plane, there should be no traditional magnetoresistance effect. The next possibility is the chiral anomaly effect. However, this behavior was observed even when the current direction was perpendicular to the magnetic field, excluding the possibility of a chiral anomaly. This excludes the possibility of the current jetting as well, since it has the similar angular dependence to the chiral anomaly^41^. Furthermore, at a temperature slightly above *T*_*c*_, negative magnetoresistance is no longer observed. Instead, there is small positive magnetoresistance (∼1%) at low fields that becomes almost constant at high fields. Thus, the negative magnetoresistance should be understood as the enhancement of superconductivity by the magnetic field.

**Figure 2:**
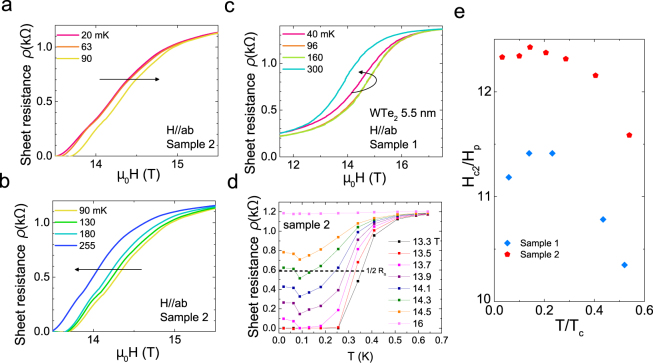
Field-induced enhancement of *T*_*c*_ at high in-plane field and low temperature. (**a**,**b**) Sheet resistance of the 5.5 nm thick WTe_2_ film (Sample 2). The black arrow marks the direction of increasing temperature. Similar non-monotonic behavior is confirmed in Sample 2. (**c**) Sheet resistance of the 5.5 nm thick WTe_2_ film (Sample 1) with *H* parallel to the film *ab*-plane. As shown by the black arrow marking the direction of increasing temperature, the upper critical field *H*_*c*2_ increases first and decreases at warmer *T*, which indicates the non-monotonic temperature *T* dependence of *H*_*c*2_. (**d**) At finite in-plane fields close and below *H*_*c*2_, the R vs. T of WTe_2_ Sample 2 shows a non-monotonic behavior, indicating the re-entrance of the superconducting state from 0.1 < *T* < 0.2 K in the *H* = 13.7 T trace. The dashed line shows the half value of the normal state resistance. As explained in the Method part, this value is used to determine *H*_*c*2_ at fixed *T*, or *T*_*c*_ at fixed *H*. (**e**) The temperature dependence of in-plane upper critical field for 5.5 nm WTe_2_ thin film around zero temperature. Both of samples show a drop of *H*_*c*2_ (2% for sample 1, 0.8% for sample 2) as T goes to zero.

Another unconventional feature is a non-monotonic behavior of *H*_*c*2_ vs *T* in the zero temperature limit. Figure 2 shows detailed data of the resistivity as a function of in-plane magnetic field and temperature at high fields. In Fig. 2(a), the *H*_*c*2_ from sample 1 at 96 mK and 160 mK is 0.29 T higher than that at 40 mK, then drops by 0.8 T at 300 mK. This non-monotonic behavior is more clear in sample 2. In Fig. 2(b), from 20 mK to 90 mK *H*_*c*2_ monotonically increases with temperature and reaches a maximum at 90 mK with *H*_*c*2_ 0.11 T higher than at base temperature. Above 90 mK, *H*_*c*2_ decreases monotonically with temperature, as shown in Fig. 2(c).

The non-monotonic behavior of *H*_*c*2_ vs *T* could be clearly seen in resistivity vs temperature plot at fixed fields from sample 2 shown in Fig. 2(d). At low field such as *H* = 13.5 T, the *R* − *T* curve shows a typically superconducting transition from a zero-resistance state at *T* = 0 to a finite normal state resistance at *T T*_*c*_. As *H* increases, however, an unconventional feature appears. As shown for the 13.7 T curve, the sample is resistive at *T* = 0, becomes non-resistive as *T* goes close to 100 mK, and eventually comes back to the resistive normal state at *T T*_*c*_. This behavior demonstrates a re-entrance of the superconducting state at finite *T* under an in-plane magnetic field.

The evolution of this re-entrance behavior leads to the non-monotonic *T* dependence of *H*_*c*2_. At 14.3 T the curve crosses the 50% of resistivity in the normal state, indicating the non-monotonic *T* dependence of *H*_*c*2_.

Figure 2(e) summarizes this unconventional behavior as *H*_*c*2_ of both samples flattens out as the temperature approaches zero then drops slightly at around 0 K. For sample 1, *H*_*c*2_/H_*p*_ enhancement reaches a maximum of 2% at 96 mK. For sample 2, *H*_*c*2_/H_*p*_ is 0.8% higher at 90 mK than at the base temperature (20 mK). This non-monotonic behavior indicates that the enhancement of *H*_*c*2_ is due to the temperature. This is the first time that field-induced enhancement of superconductivity has been observed at both zero temperature and *T*_*c*0_.

We point out that the non-monotonic behavior of *H*_*c*2_ vs *T*_*c*_ is intrinsic and does not originate from an artificial effect. First, during the measurement the samples are immersed in the He3/He4 superfluid mixture in a dilution refrigerator. This eliminates the possibility of an error arising from a temperature inhomogeneity across the samples. Second, since the in-plane *H*_*c*2_ from the thin film would be very sensitive to a field misalignment, it is necessary to adjust the angle carefully before each field sweep. Even the thermal expansion of the system could change the angle enough to affect the measurement. Thus, during the measurement at low temperature, field orientation is aligned within 0.05 degrees to the film plane at each temperature to eliminate the possibility of an angle misalignment. For each curve, the magnetic field was swept up and down to confirm that there is no angle misalignment induced during the measurement. Also, we confirmed that the magnetoresistance curves from field up-sweep and down-sweep overlap each other at each temperature. This indicates that there is no significant instability over time during the data acquisition. Furthermore, at high temperature, a field-calibrated thermometer was used to ensure that the non-monotonic behavior was not simply an artifact of the thermometer’s magnetoresistance. Thus, we conclude that the non-monotonic behavior is intrinsic.

**Figure 3:**
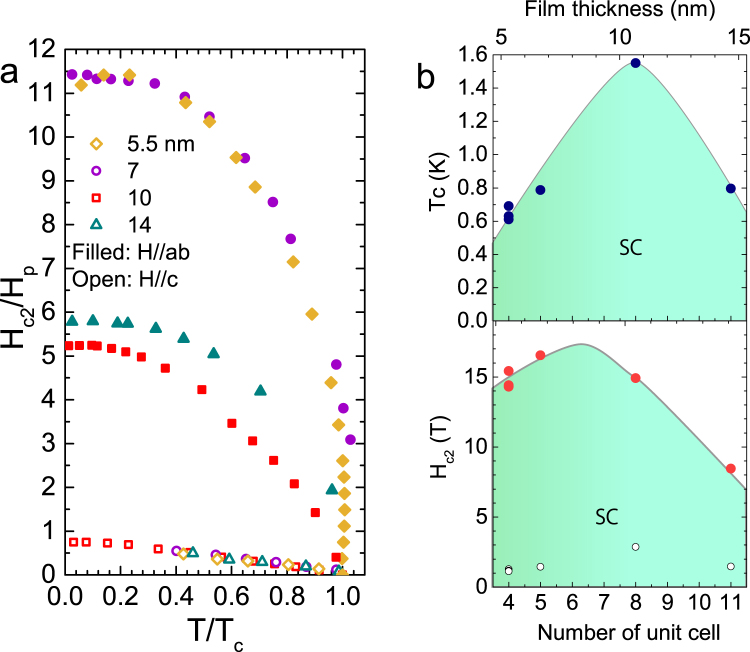
Thickness dependence of upper critical field and critical temperature. (**a**) Temperature dependence of critical field ${H}_{c2\parallel ab}$ and ${H}_{c2\parallel c}$ for 5.5, 7, 10 and 14 nm samples. The *T*_*c*_ values are normalized by the zero field transition temperature *T*_*c*0_. The *H*_*c*2_ values are normalized by the paramagnetic Pauli limit *μ*_0_*H*_*p*_ = 1.84*T*_*c*0_. (**b**) The thickness dependence of the superconducting transition temperature (top) and the in-plane upper critical field ${H}_{c2\parallel ab}$ (red circle) as well as the Pauli limit *H*_*p*_ (black circle) (bottom) at base temperature T = 20 mK. The solid lines are drawn for guidance to eye.

Finally, the thickness dependence of critical field and critical temperature is measured for thicker samples. The *T* dependence of *H*_*c*2_(T) (normalized by the paramagnetic limit *H*_*p*_) with H||ab direction for 5.5, 7, 10 and 14 nm samples is plotted in Fig. 3(a). As seen in the phase diagram, for 5.5 nm and 7 nm samples *H*_*c*2_ is more than ten times larger than the Pauli limit which is greater than any other material except for triplet and non-centrosymmetric superconductors. For the 7 nm sample, in-plane *H*_*c*2_/H_*p*_ is higher than eleven, similar to 5.5 nm sample. However, the 10 nm and 14 nm samples show much smaller *H*_*c*2_/H_*p*_, though they still exceed Pauli limit by far. On the other hand, when the magnetic field is applied parallel to *c*-axis, all *H*_*c*2_ curves overlap with each other and flatten out below Pauli limit H_*p*_. This suggests that *H*_*c*2_ is determined by the orbital limit when the field is applied perpendicular to the film surface. Figure 3(b) shows the thickness dependence of the critical temperature (top) and the critical field (bottom) at base temperature. While the 10 nm sample shows the highest *T*_*c*_, the 5.5 nm, 7 nm and 10 nm samples show similar *H*_*c*2_.

## Discussion

To our knowledge, no superconducting transition has been reported in bulk or thin film WTe_2_ at ambient pressure. When high hydrostatic pressure is applied to the crystal, several groups have reported pressure-induced superconductivity^30,31^, in which *H*_*c*2_ does not show the large enhancement beyond the paramagnetic limit. However, while the lattice constant of the crystal shrinks under high pressure, the WTe_2_ thin film experiences tensile strain and in-plane lattice expansion due to the larger lattice constant of the sapphire substrate, which is about 4.76 Å. Also, it has been reported that the pressure-induced superconductivity in WTe_2_ is associated with the structural transition from a non-centrosymmetric T_*d*_ crystal structure to a centrosymmetric 1 T’ structure^42^.

We believe that something very different is occurring in our thin film samples, because X-ray scattering suggests that the crystal structure is in the non-centrosymmetric T_*d*_ phase, as shown in the supplement. This is in contrast with the case of WTe_2_ films grown on Bi_2_ and MoS_2_ where 1 T’ phase was observed^43^. If the system is in the non-centrosymmetric T_*d*_ phase, the high *H*_*c*2_/*H*_*p*_ could be attributed to Ising type spin-orbit coupling.

We note that the electrical transport properties above *T*_*c*_ demonstrate that the epitaxially grown WTe_2_ films have electron-type carriers and that they are a heavily doped two-dimensional electronic system with the strong spin-orbit coupling (see the supplement). The crystal structure of our thin films implies that they have the same electronic structure as bulk WTe_2_ with tilted Weyl fermions. However, photoemission studies would reveal directly the energy dispersion in our MBE thin films. Our result calls for detailed tunneling and photoemission on this new family of the TMD superconducting films.

The most exciting observation is the magnetic-field-enhanced and non-monotonic superconductivity in our WTe_2_ thin films. One possible explanation is the Ising superconductivity in which the breaking of inversion symmetry predicts a non-monotonic *H*_*c*2_ vs. *T* trend near the ground state. As shown in the supplement, the competition of valley-degeneracy, the Rashba interaction, and magnetic Zeeman energy not only leads to *H*_*c*2_ much larger than the paramagnetic Pauli limit, but also leads to non-monotonic *T* dependence of *H*_*c*2_. However, also note that the simple fitting including the competition of Rashba and Zeeman terms does not explain our results as shown in the supplement.

Indeed, however, since our samples are very thin and have lattice mismatch between the film and the substrate, it is difficult to exactly determine if the samples are non-centrosymmetric or not. Thus, further experiments are necessary to confirm if the exotic non-monotonic behavior of *H*_*c*2_ as well as extremely high *H*_*c*2_/*H*_*p*_ are related to the symmetry. These experiments may further help determine the implication of the pairing symmetry. If the sample is centrosymmetric and in the 1 T’ phase, high *H*_*c*2_/*H*_*p*_ might arise from the p-wave pairing. We note further that even in the centrosymmetric 1 T’ bulk phase, the thin film is still non-centrosymmetric when an odd number of atomic layers are grown.

The other possibility is that finite-momentum pairing theoretically predicts a non-monotonic ${H}_{c2\parallel ab}-T$ trace^3^, an interesting state where the in-plane magnetic field enhances *T*_*c*_. This exotic pairing state may be enabled in WTe_2_ by inversion symmetry breaking and the novel type-II Weyl semimetal electronic state in WTe_2_. We note that generally this may lead to the magnetic field enhancement both near the ground state and near the zero-field superconducting transition at *T*_*c*0_.

In summary, we resolved unconventional superconducting behaviors of MBE grown WTe_2_ thin films. We observed a 1.6% enhancement of *T*_*c*_ by magnetic field, non-monotonic *H*_*c*2_ vs *T*_*c*_ in the zero temperature limit, and an *H*_*c*2_ more than 10 times larger than the Pauli limit *H*_*p*_. These results not only support the existence of Ising superconductivity, but also indicate further unconventional properties.

## Methods

### Thin film growth

WTe_2_ thin films were grown on a sapphire substrate using a Veeco Genxplor MBE system. Prior to loading into the MBE chamber, the *c*-plane sapphire substrates were first cleaned using acetone, methanol, and deionized water. The sapphire was subsequently cleaned at elevated temperatures in the MBE chamber prior to growth initiation. During the growth of WTe_2_, the substrate temperature was ∼350°. A PBN (Pyrolytic Boron Nitride) effusion cell and an e-beam evaporator were used for the thermal evaporation of Te and W, respectively. The Te flux was measured to be ∼5 × 10^−8^ torr. The growth rate was estimated to be ∼1.2 Å/min. A very slow deposition rate is used to reduce the formation of Te vacancies, which has been commonly observed for transition metal telluride materials^44^.

### Scanning probe microscopy

The surface morphology of as-grown WTe_2_ films were determined by scanning probe microscopy (SPM, Bruker MultiMode) using the tapping mode under ambient conditions. The probe was coated with Cr/Pt thin film with a force constant of 40 N/m, and the tip radius was less than 25 nm.

### X-ray photoelectron spectroscopy

X-ray photoelectron spectroscopy (XPS, Thermo Sci.) was employed to investigate the element components, bonding structure, and surface stability of WTe_2_ thin films. The X-ray source is Al-*Kα* and has a spot size of 400 *μ*m. Survey scans were performed from 0 to 1350 eV for the binding energy, and core-level scans were from 235 to 270 eV for W 4d and from 560 to 600 eV for Te 3d, respectively.

### Transmission electron microscopy

High-resolution transmission electron microscopy (HR-TEM, JEOL 2100 F) revealed cross-sectional atomic structure of WTe_2_ thin films, and the element distribution was studied using an energy dispersive X-ray spectroscope (EDX, Oxford Ins, AZtec). The specimen was prepared using focused ion beam (FIB) technique (Hitachi, FB2000A) with a titanium protection layer on the top surface. This preparation method made WTe_2_ films intact, preserved the surface morphology, and revealed the interface heterostructure between WTe_2_ and sapphire.

### X-ray Diffraction

Crystal structures of MBE-grown WTe_2_ thin films were analyzed by X-ray diffraction (XRD, Bruker D8 Advance) in the Bragg-Brentano geometry. The X-ray source is Cu-*Kα* with a wavelength of 1.542 Å. Diffraction spectra were collected from 10° to 80° (2theta) with a step size of 0.02°.

### Electrical transport characterization

The resistance of WTe_2_ thin films was measured by standard four-probe measurement in Oxford Instruments Triton 200, Quantum Design PPMS and National High Magnetic Field Laboratory(NHMFL) using Keithley 6221 AC current source (typically around 13 Hz) and Stanford Research SR830 lock-in amplifier. In NHMFL, high magnetic fields up to 35 T were applied by the resistive magnet. Small enough excitation of current was applied so that we can ignore the effect of heating or H_*c*2_ suppression. The current dependence of voltage is obtained by the combination of Keithley 6221 and 2182 A. The critical temperature of superconducting transition T_*c*_ is defined as *R*(*T*_*c*_) = 0.5*R*(*T* = 4*K*). As the magnetoresistance becomes saturated at high fields, critical field *H*_*c*2_ is defined as well by *R*(*H*_*c*2_) = 0.5*R*_*sat*_, where *R*_*sat*_ is the saturated resistance. Critical current *I*_*c*_ is determined as *R*(*I* = *I*_*c*_) = 0.5*R*(*I* = 1*mA*) obtained from the I-V curves.

## Electronic supplementary material

Supplemental Materials
