# Supplementary material for: Magnetic Field Enhanced Superconductivity in Epitaxial Thin Film WTe2
Source: Sci Rep. 2018 Apr 25;8:6520. doi: 10.1038/s41598-018-24736-x (PMC5917024; doi:10.1038/s41598-018-24736-x)
Supplement: Supplementary file 1 — Supplemental Materials [file 41598_2018_24736_MOESM1_ESM.pdf]

Magnetic Field Enhanced Superconductivity in Epitaxial Thin Film WTe_2

Tomoya Asaba[1†], Yongjie Wang[2†], Gang Li[1], Ziji Xiang[1], B.J. Lawson[1], Colin Tinsman[1], Lu Chen[1], Shangnan Zhou[1], Songrui Zhao[2], David Laleyan[2], Yi Li[3], Zetian Mi[2], Lu Li[1⋆1]

[11]*Department of Physics, University of Michigan, Ann Arbor, MI 48109, USA*

[2] *Department of Electrical and Computer Engineering, McGill University, Montreal, Quebec H3A 0E9, Canada*

[3] *Department of Physics and Astronomy, Johns Hopkins University, Baltimore, MA, USA*

(Dated: March 21, 2018)

SUPPLEMENTAL MATERIALS

Magnetic field dependence of the resistivity in WTe_2 films of various thickness

To reveal the intrinsic nature of the superconducting state of WTe_2 thin films, we carried out the magnetotransport studies on samples with various thicknesses. Fig. S1 and Fig. S2 show the sheet resistance of the 7,10 and 14 nm thick WTe_2 films with two magnetic field geometries: H parallel to the film's ab-plane and c-axis. H_{c2} are determined following the same analysis as shown in the Materials and Methods parts.

We further studied the magnetoresistance with a field H applied parallel to c-axis. Fig. S2 shows the sheet resistance ρ of the 5.5, 7, 10 and 14 nm thick WTe_2 films. All of the films show much smaller H_{c2} than with H parallel to ab-plane.

Weak anti-localization in the WTe_2 thin films

The electrical transport measurements above T_c demonstrate that these WTe_2 films are two-dimensional with strong spin-orbit-coupling. Fig. S3(a) shows the magneto-resistance (MR) of the same film in Fig. S6(a) at different temperatures above T_c. The conductivity of WTe_2 film is enhanced greatly near zero field at low T. This suggests a weak anti-localization (WAL) in the epitaxial WTe_2 films. When WAL occurs, the destructive interference between time-reversed electron paths lowers the resistivity. In the low mobility regime, WAL negative correlation in conductance is described by Hikami-Larkin-Nagaoka (HLN) theory [1]:

$$\Delta\sigma_{xx}(H) = \alpha\frac{e^2}{2\pi^2\hbar}\left[\ln\left(\frac{B_\phi}{B}\right) - \psi\left(\frac{1}{2}+\frac{B_\phi}{B}\right)\right] \tag{S1}$$

where ψ is the digamma function, $B_\phi = \frac{\hbar}{4eL_\phi^2}$ with L_ϕ as phase dephasing length, and $\alpha = \frac{1}{2}$ for each conduction channel. To fit the curve, α is fixed to 1.16 obtained from the fitting at 5 K. Shown as red dashed lines in Fig. S3(a), the fitting based on Eq. S1 agrees with the experimental results. The value of α =1.16 is close to 1, consistent with two conducting channels from the top and bottom surface. This feature is observed in thin flake WTe_2 [2] as well as in TIs such as Bi_2Se_3 [3] or Bi_2Te_3 [4]. The temperature dependence of B_ϕ and L_ϕ are shown in Fig. S3 (b).

This WAL behavior confirms the strong spin-orbit coupling. The logarithmic behavior also suggests the electronic system in our WTe_2 films are two-dimensional.

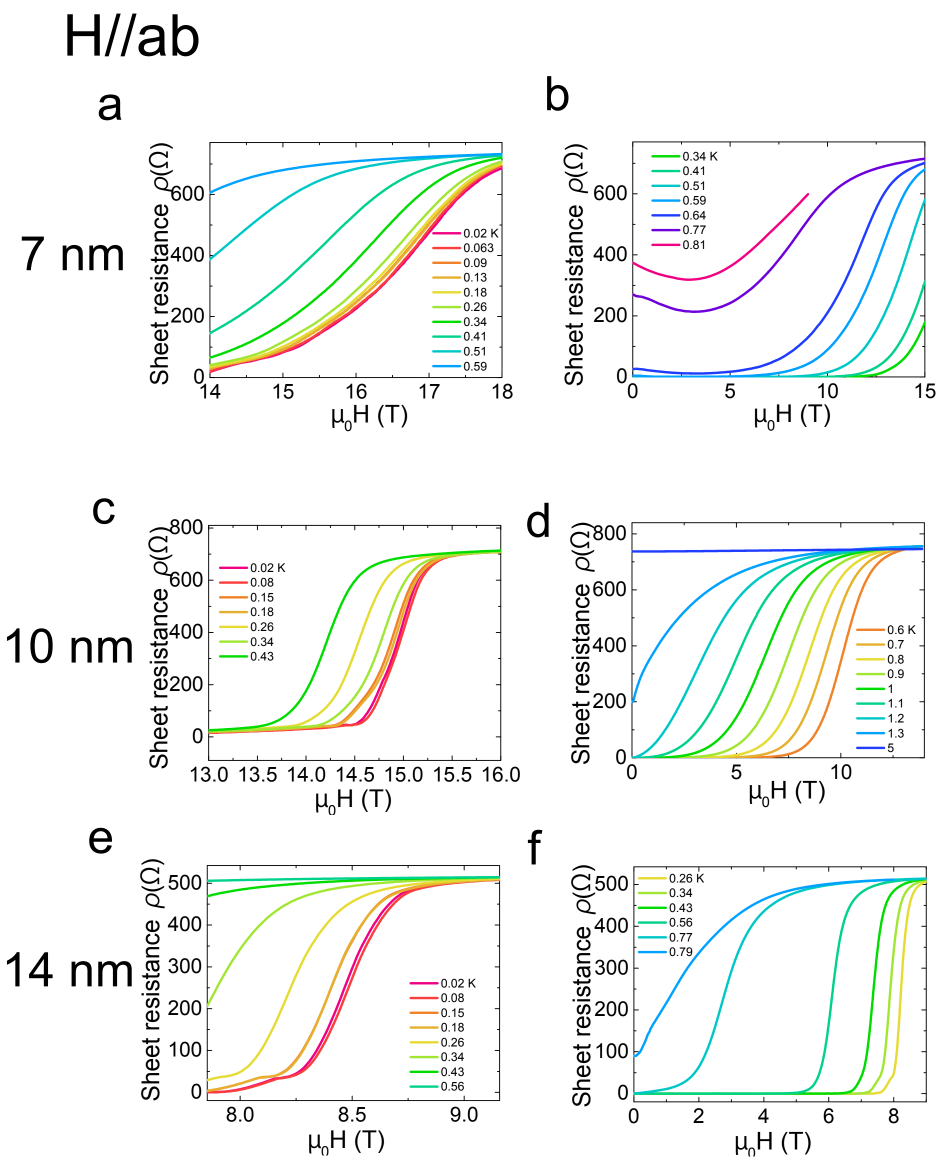

FIG. S1. **Magnetic field H dependence of the sheet resistivity ρ of 7 nm, 10 nm and 14 nm thick WTe_2 films as the H parallel to the film plane.** (a) H-dependence of ρ of the 7 nm film when H is parallel to the film *ab* plane. (b) Expansion of (a) at low fields and high temperatures. (c)-(f) The same sets for the 10 and 14 nm thick WTe_2 films.

We note that the WTe_2 films do not show the large non-saturating MR as shown in the bulk crystals of WTe_2 [5]. The large non-saturating MR was believed to be a result of the almost perfect balancing between the electron and hole pockets in the bulk WTe_2 [6, 7]. Our result suggests that the electronic structures in our epitaxial WTe_2 films are far from the electron-hole balancing. Indeed, the Hall effect on these films demonstrate that our

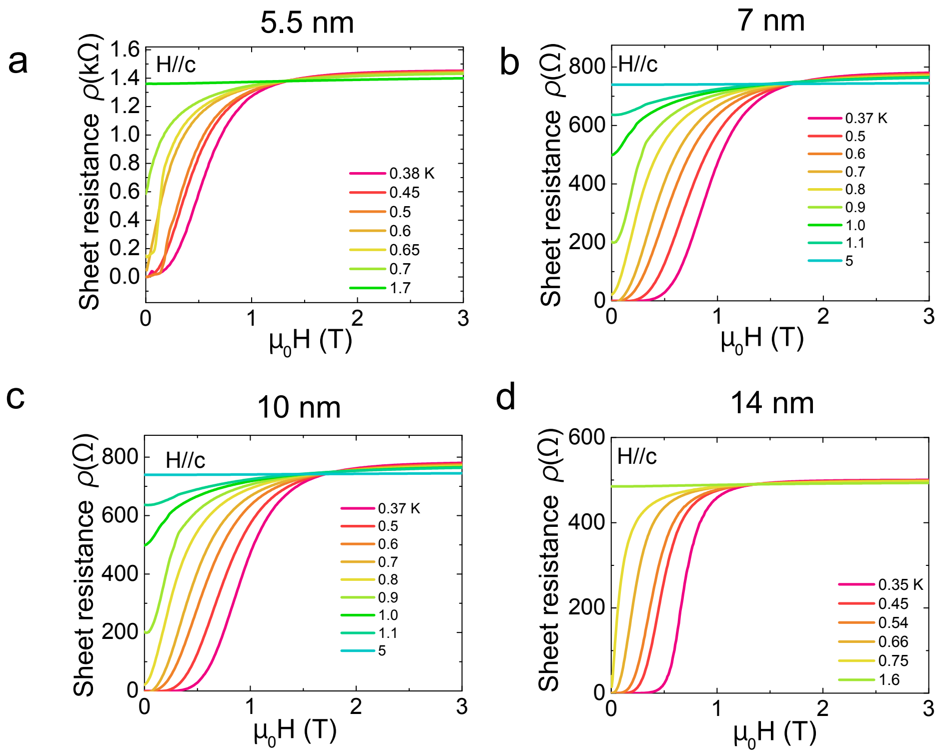

FIG. S2. **Magnetoresistance of WTe_2 films with field H applied parallel to the c-axis.** For the thickness of (a) 5.5 nm, (b) 7nm, (c) 10nm and (d) 14 nm..

WTe_2 films are heavily doped. Fig. S3(c) shows the Hall effect curves of the same film presented in Fig. S6(c). The sign of the Hall effect shows the carriers are electrons. The Hall signal is almost linear up to 14 T and only one electron carrier is resolved. Calculated sheet carrier density is as high as 3.3×10^{15} cm^{-2}, which corresponds to about 2 electrons per 1 tungsten atom. In summary, the electrical transport properties above T_c demonstrate that the epitaxially grown WTe_2 films are heavily doped two-dimensional electronic systems with strong spin-orbit coupling.

Control experiment of tungsten thin films

Control experiments were also carried out to assure the superconductivity is observed in the crystalline WTe_2 films, not from the impurity superconducting phase of W [8]. We compared the T dependence of the sheet resistance of 10 nm thick W films grown in the exact same MBE chamber on the same type of sapphire substrates. No superconductivity was observed down to 25 mK, the lowest temperature that we can reach with our dilution

refrigerators (Fig. S4). This control experiment, together with the clean crystal structure of the WTe_2 films and the thickness dependence of the superconducting T_c, indeed suggests that the superconductivity comes from the epitaxial WTe_2 thin films.

Mutual inductance measurement of WTe_2 film

The mutual inductance technique was used to confirm the superconductivity in the thin film. The temperature dependence of the voltage signal is measured at both zero field and 1 T to compare the signal from the superconducting and normal state. The field is applied perpendicular to the film to suppress the superconductivity. As shown, at zero field diamagnetic screening (Meissner effect) was observed below 2 K while there is almost no temperature dependence when 1 T was applied (Fig. S5). Combined with the zero resistance observed, we confirmed the superconductivity in the film.

Surface, structure and electrical properties of molecular beam epitaxially grown WTe_2 thin films on sapphire

Structural properties of WTe_2 films were studied using cross-sectional transmission electron microscopy (TEM) (see Method). Shown in Fig. S6(c) is the low-magnification TEM image, suggesting the formation of uniform and continuous film structure directly on a sapphire substrate without the presence of pinholes. The unwrinkled interface between WTe_2 and Ti further confirms the smooth surface of WTe_2 thin films, which is consistent with the SPM measurement shown in Fig. S6(a). The thickness is determined to be $\sim$ 10 nm. Illustrated in Fig. S6(d) is the high-resolution TEM image for the dashed green box in Fig. S6(c), which shows a distinct layered structure, with the atomic planes parallel to the sapphire/WTe_2 interface. The interplanar spacing along the growth direction is derived to be 0.667 nm, which is slightly smaller than that along the *c*-axis (0.7035 nm) of bulk WTe_2, suggesting the presence of tensile strain in the epitaxial WTe_2 films on sapphire. In the Td structure, the interplanar spacing is half of the lattice constant along the c-axis. The energy dispersive X-ray spectroscopy (EDX) scans were performed along the solid blue line in Fig. S6(c). The elemental distribution, including Al, O, W, Te and Ti (the top protection layer used in sample preparation) are shown in Fig. S6(e). It is seen that W

and Te signals dominate for regions corresponding to the WTe_2 layer. The formation of an abrupt WTe_2/sapphire heterointerface is also evident based on the sharp increase of W and Te signals. Moreover, the W and Te signals remain relatively constant in the WTe_2 layer, suggesting uniform compositional distribution along the growth direction.

Structural characterization of WTe_2 films were further performed using X-ray diffraction (see Method). Shown in Fig. S6(f) is the theta-2theta scan of a 10 nm WTe_2 film on sapphire. Multiple peaks corresponding to diffraction from the (024) and (122) planes can be clearly identified, which provides strong evidence that the epitaxial WTe_2 film is of T_d structure, rather than 2H phase. The approximate space group of WTe_2 crystals is $Pmn2_1$ with an orthorhombic structure. The tungsten atoms locate in an off-center position of distorted tellurium octahedral units [5, 9–11]. In spite of the extremely thin layer, the linewidth is measured to be less than 0.2°, which demonstrates excellent crystallinity of MBE-grown WTe_2 thin films. Additionally, diffraction peaks corresponding to the (004), (113), (109), (231), and (233) planes are detected, which are consistent with theoretical and experimental studies of single crystal WTe_2 in the T_d phase [9, 11, 12].

Transport properties of several samples were measured down to 0.3 K. All of the samples are conductive and display superconductivity at low temperature around 1 K. An example of the temperature T dependence of resistivity ρ is shown in Fig. S6(g). The film resistivity ρ increases slowly as T decreases from room temperature to 50 K. Below 50 K, ρ is independent of T until ρ drops to zero at $T_c = 1.3$ K. ρ drops more than 3 orders of magnitude to below the measurement noise limit. This sharp drop of resistivity suggests that the epitaxial WTe_2 film turns superconducting at the ground state. The superconducting transition temperature T_c is defined as $R(T_c) = 0.5R(T = 4K)$. The width of the superconducting transition is quite narrow, about $\sim$ 80 mK, which is determined by the width of 20 % and 80 % of ρ(4K). A similar superconducting transition is observed at $T_c = 0.7$ K and $T_c = 0.6K$ in the 5.5 nm thick epitaxial WTe_2 films. This sharp transition suggests the uniformity of the superconducting phase of our epitaxial films.

Angular dependence of upper critical field of WTe_2 thin films

Fig. S7 (a) shows the magnetoresistance at the 10 nm epitaxial WTe_2 film at the base temperate 300 mK. The magnetic field H is applied at various tilt angles, ϕ, which is defined

as the angle of the H field away form the normal axis (c-axis) of the film. The upper critical field H_{c2} is determined as 50 % of the constant resistivity value at high fields. Fig. S7 (b) summarizes the angular dependence of H_{c2} with the fit from Eq. S2 [13]:

$$\frac{H_{c2}(\phi)}{H_{c2\|c}}\cos\phi + \left(\frac{H_{c2}(\phi)}{H_{c2\|ab}}\sin\phi\right)^2 = 1 \tag{S2}$$

The sharp change near $\phi = 90°$ demands that our study of the angular dependence needs a stable and precise control of the magnetic field orientation relative to the thin film samples.

2D superconductivity in WTe_2 thin film

We observed characteristic current-voltage feature of the 2D superconducting state. Fig. S8(a) shows the current I dependence of voltage V at different temperature T in logarithmic scale. As I increases from zero, V starts at zero within noise until I reaches critical current I_c. Fig. S8(b) shows the T dependence of the critical current I_c. The maximum I_c is 2.8 mA/cm at 30 mK. The observation of critical current confirms the superconductivity in the ground state of epitaxial WTe_2 film.

More importantly, the V vs. I curve indicates the low dimensionality of our samples. If the system is 2-dimensional, the transition into superconductivity is a BKT transition [14, 15]. Through the BKT transition, vortex-antivortex pairs unbind, leading to the power-law behavior of V vs. I [16–18]. At the BKT transition temperature T_{BKT}, $V \propto I^{\alpha}$ with exponent $\alpha = 3$. As shown in Fig. S8(a), the sample clearly shows $V \propto I^{\alpha}$ behavior at around the superconducting transition. The exponent of the power-law dependence is tracked and shown in Fig. S8(c). At $T = 1.26$ K the exponent, α, becomes close to 3, indicating that this temperature is T_{BKT}. The observation of the non-linear I-V behavior confirms the low dimensionality of the electronic system in the WTe_2 films.

Ising superconductivity fitting of 5.5 nm WTe_2 thin films

We tried to fit H_{c2} vs T_c curves using theoretical model based on the Ising sueperconductivity [19]. The equation is following.

$$\ln\left(\frac{T_c}{T_{c0}}\right) + \Psi(\rho_-) + \Psi(\rho_+) + [\Psi(\rho_-) - \Psi(\rho_+)]\frac{(\mathbf{g_F} + \beta_{\mathbf{SO}})^2 - \mathbf{b}^2}{|\mathbf{g_F} + \beta_{\mathbf{SO}} - \mathbf{b}||\mathbf{g_F} + \beta_{\mathbf{SO}} + \mathbf{b}|} = 0 \tag{S3}$$

where

$$\rho_{\pm} = \frac{|\mathbf{g_F} + \beta_{\mathbf{SO}} + \mathbf{b}| \pm |\mathbf{g_F} + \beta_{\mathbf{SO}} - \mathbf{b}|}{2\pi T_c} \tag{S4}$$

with parameters

$$\mathbf{b} = (\mu_B B_{c2}, 0, 0), \mathbf{g_F} = (\alpha_F k_F, -\alpha_F k_F, 0), \beta_{\mathbf{SO}} = (0, 0, \beta_{SO}) \tag{S5}$$

and

$$\Psi(\rho) \equiv \frac{1}{2} Re \left[\psi\left(\frac{1+i\rho}{2}\right) - \psi\left(\frac{1}{2}\right)\right] \tag{S6}$$

The fitting base on the Ising superconductivity captures the great enhancement of the upper critical field with in-plane magnetic fields. However, the fitting curve does not fit very well with our data especially at low T. Moreover, the theory does not explain the magnetic-field enhancement of T_c.

[1] S. Hikami, A. I. Larkin, and Y. Nagaoka, Progress of Theoretical Physics **63**, 707 (1980).

[2] L. Wang, I. Gutiérrez-Lezama, C. Barreteau, N. Ubrig, E. Giannini, and A. F. Morpurgo, Nature communications **6** (2015).

[3] M. Liu, J. Zhang, C.-Z. Chang, Z. Zhang, X. Feng, K. Li, K. He, L.-l. Wang, X. Chen, X. Dai, Z. Fang, Q.-K. Xue, X. Ma, and Y. Wang, Physical review letters **108**, 036805 (2012).

[4] H.-T. He, G. Wang, T. Zhang, I.-K. Sou, G. K. Wong, J.-N. Wang, H.-Z. Lu, S.-Q. Shen, and F.-C. Zhang, Physical review letters **106**, 166805 (2011).

[5] M. N. Ali, J. Xiong, S. Flynn, J. Tao, Q. D. Gibson, L. M. Schoop, T. Liang, N. Haldolaarachchige, M. Hirschberger, N. P. Ong, and R. J. Cava, Nature **514**, 205 (2014).

[6] E. Sondheimer and A. Wilson, in *Proceedings of the Royal Society of London A: Mathematical, Physical and Engineering Sciences*, Vol. 190 (The Royal Society, 1947) pp. 435–455.

[7] S. Murzin, S. Dorozhkin, G. Landwehr, and A. Gossard, Journal of Experimental and Theoretical Physics Letters **67**, 113 (1998).

[8] W. Bond, A. Cooper, K. Andres, G. Hull, T. Geballe, and B. Matthias, Physical Review Letters **15**, 260 (1965).

[9] C.-H. Lee, E. C. Silva, L. Calderin, M. A. T. Nguyen, M. J. Hollander, B. Bersch, T. E. Mallouk, and J. A. Robinson, Scientific reports **5** (2015).

[10] B. E. Brown, Acta Crystallographica **20**, 268 (1966).

[11] D. Kang, Y. Zhou, W. Yi, C. Yang, J. Guo, Y. Shi, S. Zhang, Z. Wang, C. Zhang, S. Jiang, A. Li, K. Yang, Q. Wu, G. Zhang, L. Sun, and Z. Zhao, Nature communications **6** (2015).

[12] P. Cai, J. Hu, L. He, J. Pan, X. Hong, Z. Zhang, J. Zhang, J. Wei, Z. Mao, and S. Li, Physical review letters **115**, 057202 (2015).

[13] M. Tinkham, *Introduction to superconductivity* (Courier Corporation, 1996).

[14] V. Berezinskii, Sov. Phys. JETP **32**, 493 (1971).

[15] J. M. Kosterlitz and D. J. Thouless, Journal of Physics C: Solid State Physics **6**, 1181 (1973).

[16] M. Beasley, J. Mooij, and T. Orlando, Physical Review Letters **42**, 1165 (1979).

[17] K. Medvedyeva, B. J. Kim, and P. Minnhagen, Physical Review B **62**, 14531 (2000).

[18] N. Reyren, S. Thiel, A. D. Caviglia, L. F. Kourkoutis, G. Hammerl, C. Richter, C. W. Schneider, T. Kopp, A.-S. Rüetschi, D. Jaccard, M. Gabay, D. A. Muller, J.-M. Triscone, and J. Mannhart, Science **317**, 1196 (2007).

[19] J. Lu, O. Zheliuk, I. Leermakers, N. Yuan, U. Zeitler, K. Law, and J. Ye, Science **350**, 1353 (2015).

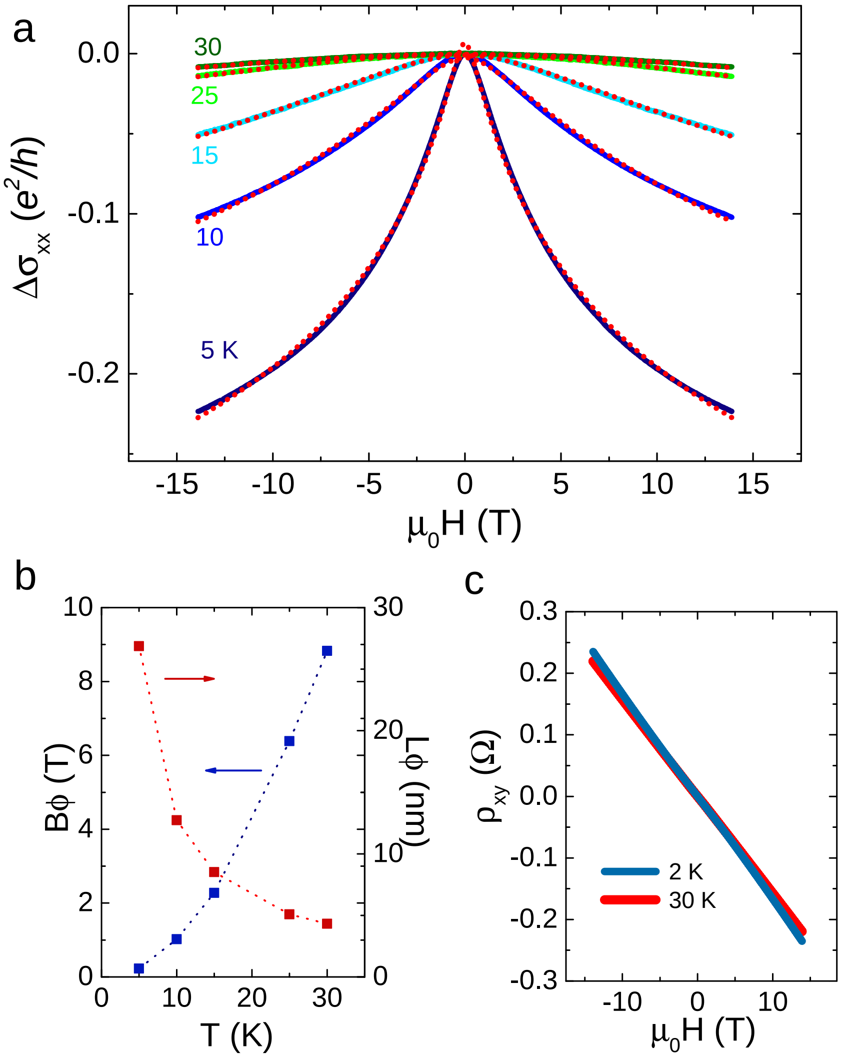

FIG. S3. **Magnetotransport properties in WTe_2 thin film.** (color online) (a). Magneto-conductance of the film at different temperatures between 5 K and 30 K showing WAL behavior. Temperature is high enough so that the effect of superconductivity can be ignored. The solid lines indicate experimental data and red dashed lines are obtained by fitting the experimental data using HLK theory described as equation (4). Experimental data is well fitted with fixed $\alpha = 1.16$, suggesting that there are two conducting channels from top and bottom surface. (b). Temperature dependence of B_ϕ and L_ϕ obtained by the fitting. (c). The 2D Hall resistivity ρ_{xy} at 2 K and 30 K. Negative sign of ρ_{xy} indicates that the carrier is electron, and only one carrier is resolved up to 14 T.

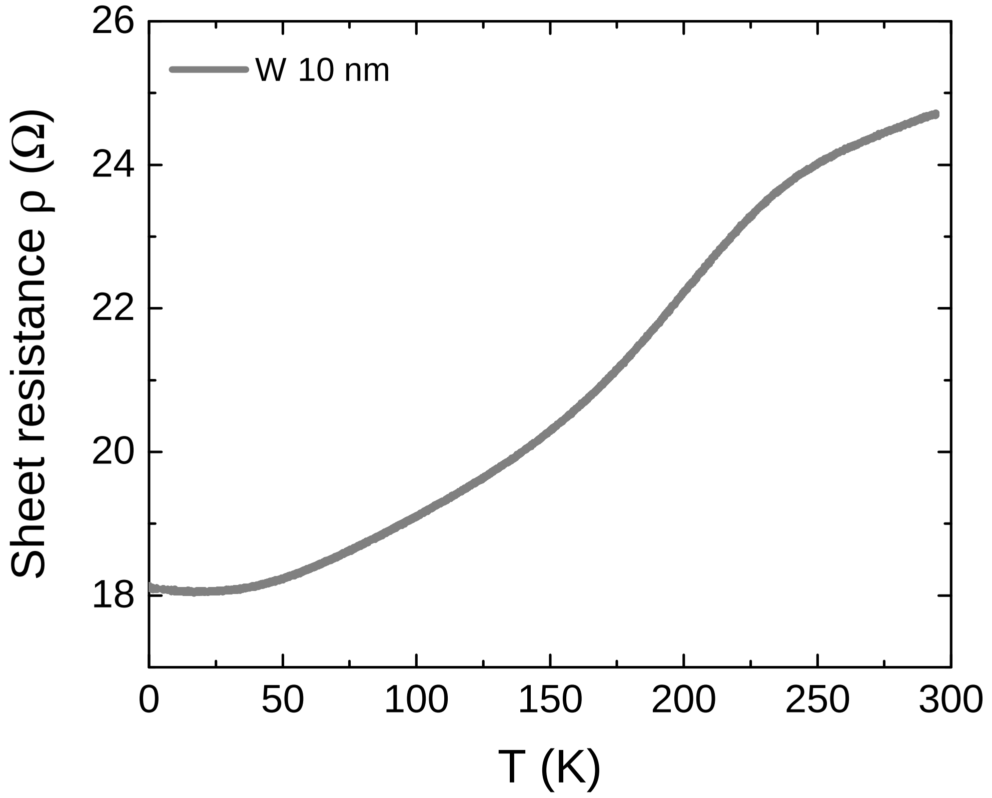

FIG. S4. **Temperature dependence of the sheet resistivity of the MBE grown 10 nm thick tungsten film.** (color online) .

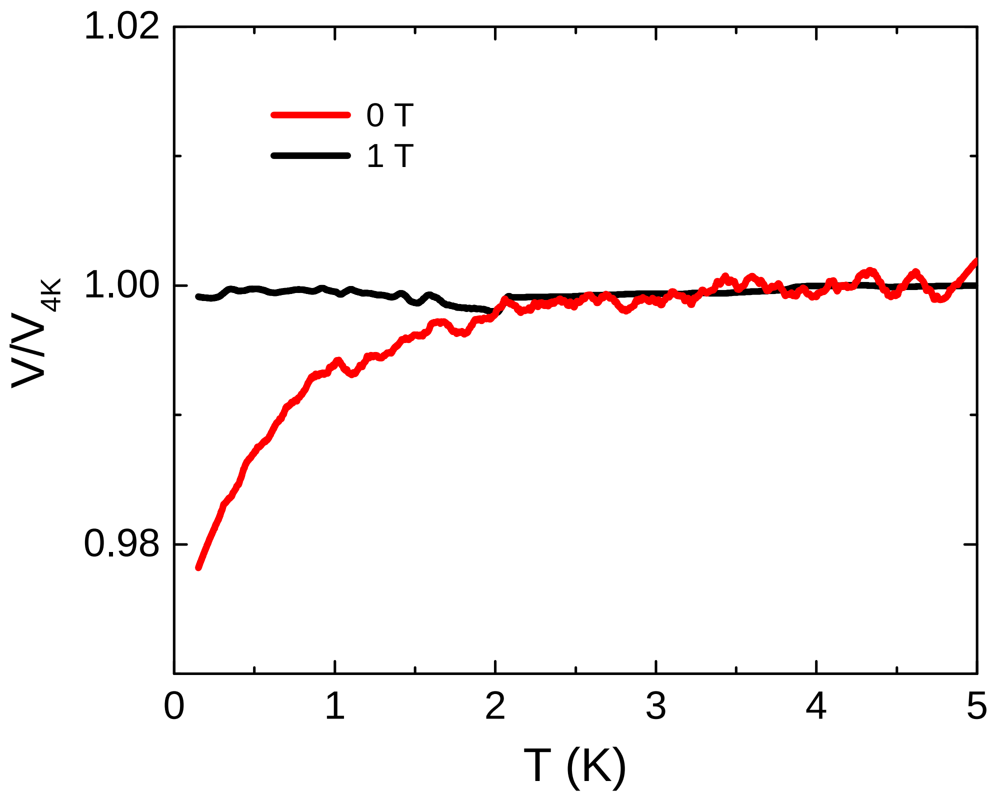

FIG. S5. **Temperature dependence of the voltage across the pickup coil showing Meissner effect at zero field.** (color online) .

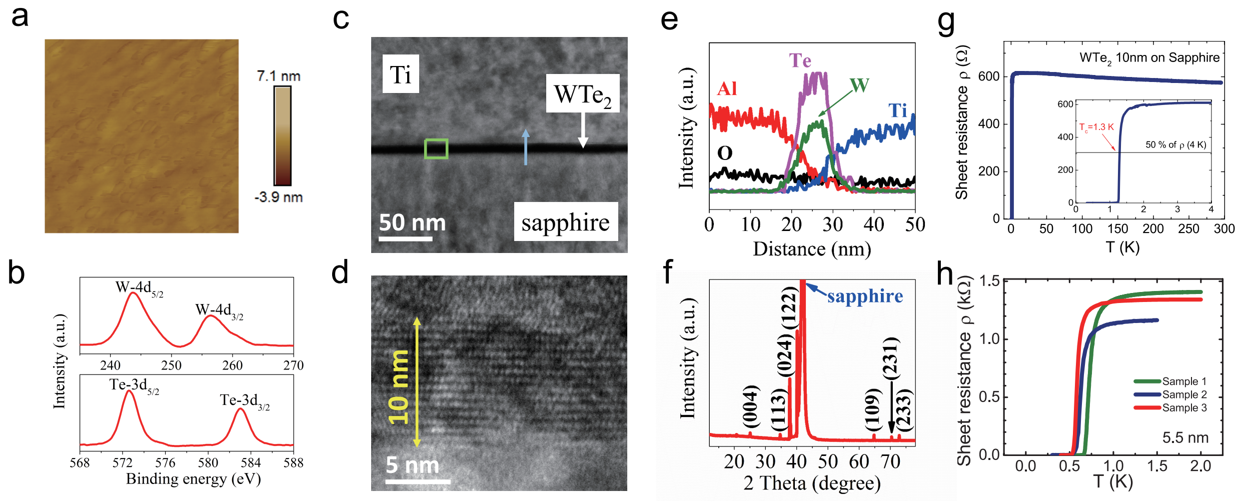

FIG. S6. **Surface, structure and electrical properties of molecular beam epitaxially grown WTe_2 thin films on sapphire.** (a).The surface topography profile measured by scanning probe microscopy demonstrating a smooth surface with a roughness of 0.22 nm. (b). High-resolution XPS core-level peaks of W (4d orbit) and Te (3d orbit). (c). Low-magnification side-view TEM image of WTe_2 film on sapphire with titanium protection layer on top. The rectangular region bordered by green dashed lines is for high-resolution TEM analysis, and the EDX line scan analysis is performed along the blue arrow. (d). High-resolution cross-sectional TEM image shows clearly layered structure with an interplanar spacing of 0.667 nm. (e). High-resolution line-scan EDX profile confirms the uniform distribution of W and Te in the WTe_2 layer. (f). X-ray diffraction by Bragg- Brentano incidence measurements with Miller indexes corresponding to Td-WTe_2 crystal structure. (g). Temperature dependence of sheet resistance of 10 nm WTe_2 thin film epitaxially grown on the sapphire substrate ranging from 0.3 K to 300 K. Inset shows the superconducting transition around critical temperature $T_c = 1.3$ K. (g). Temperature dependence of sheet resistance of the 5.5 nm WTe_2 thin films at the base temperatures. T_c is about 0.7 K.

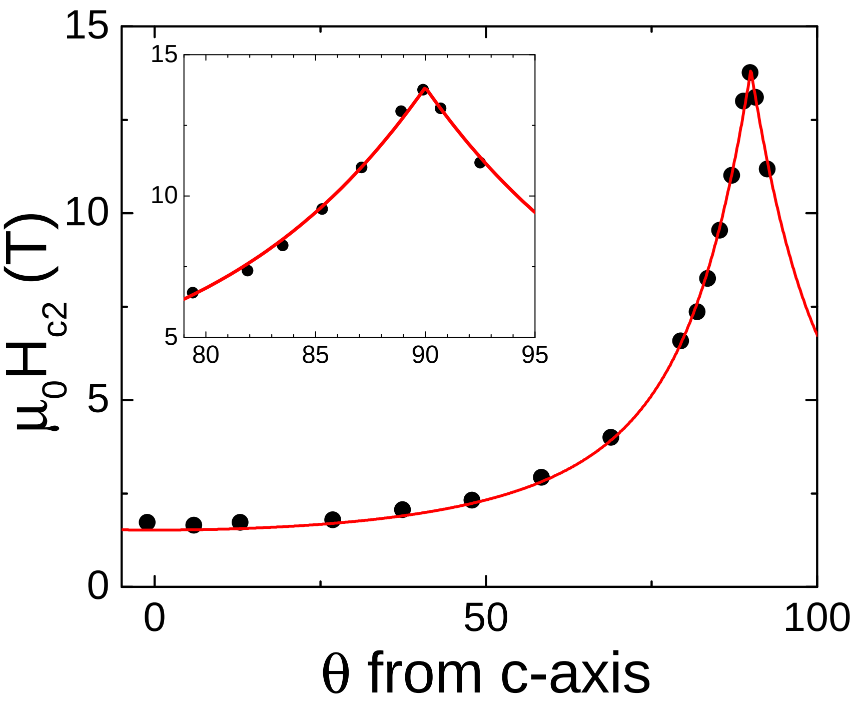

FIG. S7. **Angular dependence of upper critical field in WTe_2 thin film.** (a) The magnetic field H dependence of the 10 nm WTe_2 film sheet resistance, ρ, at the base temperature 300 mK in a number of H directions. The tilt angle ϕ is defined as the angle between the H field and the film normal axis (c-axis). The upper critical field H_{c2} is determined as the field value where R is half of the normal state sheet resistance. (b) The angular dependence of H_{c2} is perfectly consistent with the 2D behavior as in Equation S2.

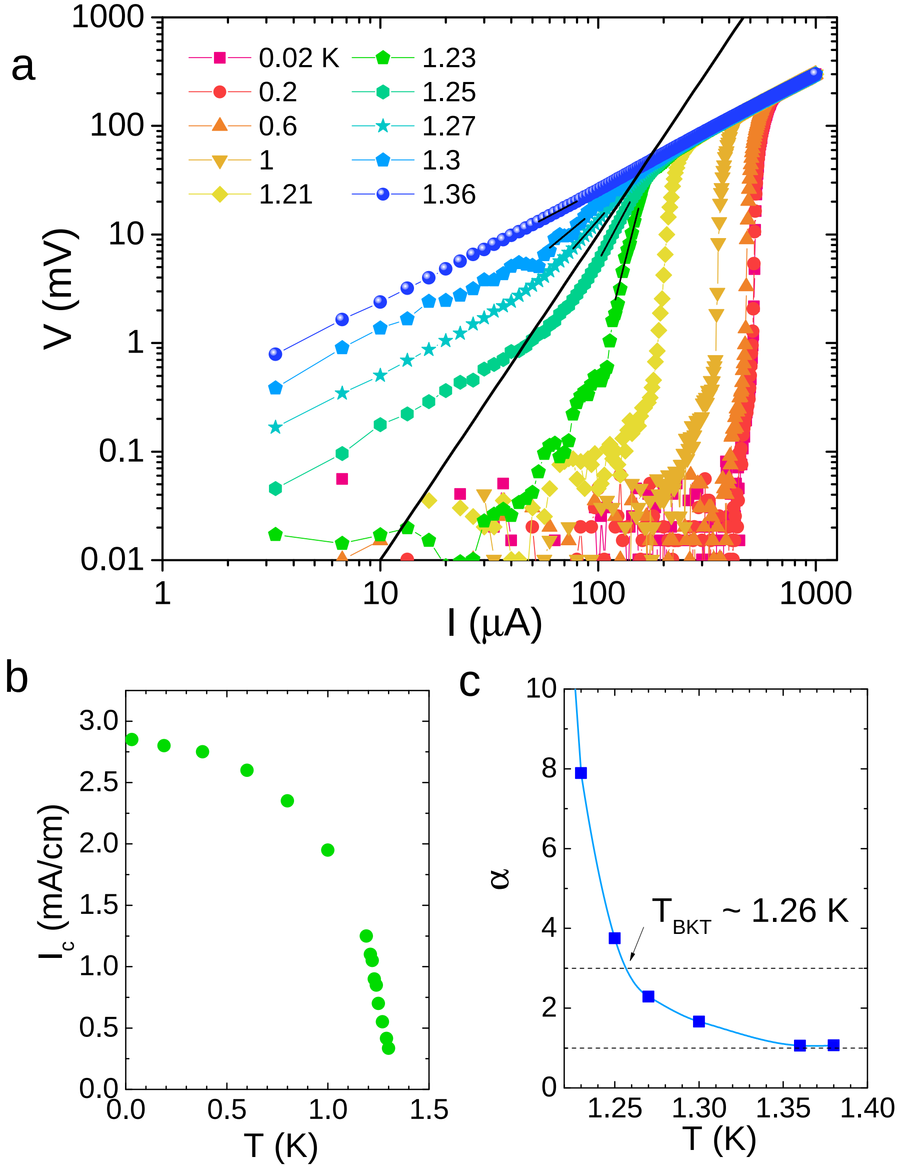

FIG. S8. **2D superconductivity in WTe_2 thin film.** (a) The voltage V vs. current I curves at around superconducting transition temperature T_c on a logarithmic scale. The short black lines represent the fitted polynomial functions at the transition. The long black line indicates $V \propto I^\alpha$ which leads $T_{BKT} \sim 1.26$ K. (b) Temperature dependence of critical current density I_c. (c) Temperature dependence of the power-law exponent α obtained from the fitting. Two dashed black lines correspond to $\alpha = 3$ and $\alpha = 1$, respectively.

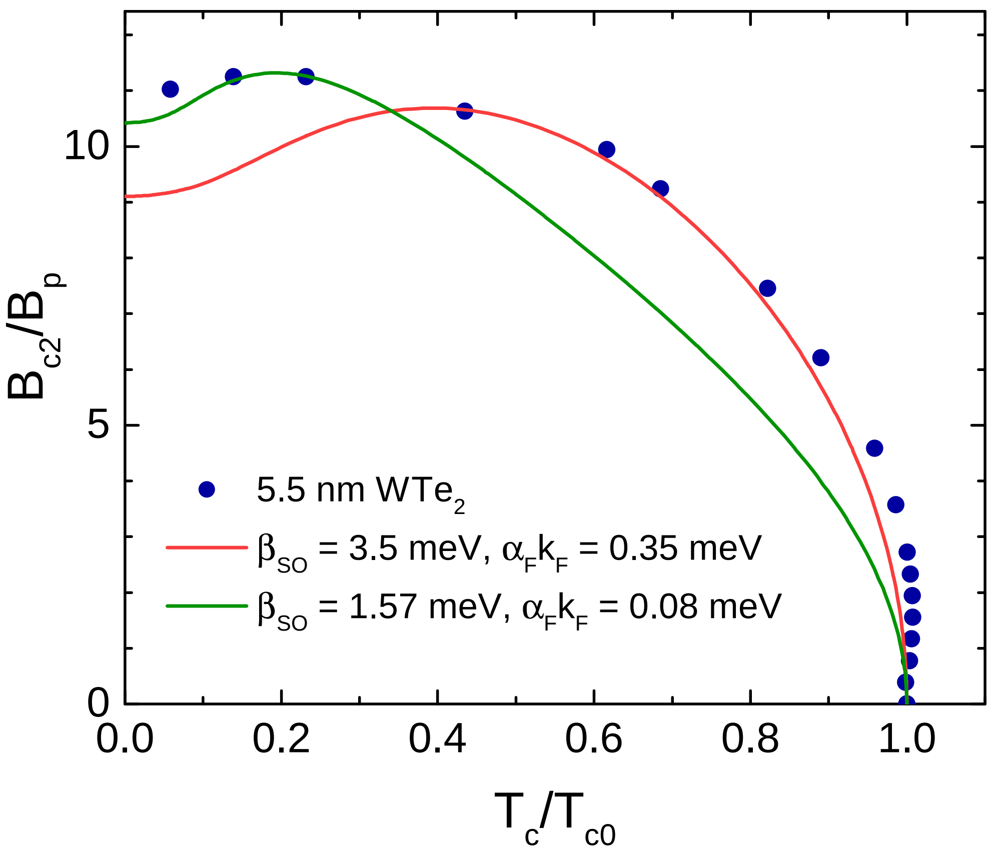

FIG. S9. **Ising superconductivity fitting in WTe_2 thin film.** The H_{c2}/H_p vs T_c/T_{c0} curve of 5.5 nm WTe_2 thin film was fit using the equation S3 based on the theory of Ising superconductivity[19]. Solid lines correspond to β_{SO} = 3.5 meV, $\alpha_f k_F$ = 0.35 meV (red) and $\beta_{SO} = 1.57$ meV, $\alpha_f k_F = 0.08$ meV (green), respectively.
